# Temporal response of the tiger salamander (*Ambystoma tigrinum*) to 3,000 years of climatic variation

**DOI:** 10.1186/1472-6785-5-7

**Published:** 2005-09-13

**Authors:** Judsen E Bruzgul, Webb Long, Elizabeth A Hadly

**Affiliations:** 1 Department of Biological Sciences Stanford University Stanford USA; 2 School of Medicine University of Vermont Burlington USA

## Abstract

**Background:**

Amphibians are sensitive indicators of environmental conditions and show measurable responses, such as changes in phenology, abundance and range limits to local changes in precipitation and temperature regimes. Amphibians offer unique opportunities to study the important ecological and evolutionary implications of responses in life history characteristics to climatic change. We analyzed a late-Holocene fossil record of the Tiger Salamander (*Ambystoma tigrinum*) for evidence of population-level changes in body size and paedomorphosis to climatic change over the last 3000 years.

**Results:**

We found a significant difference in body size index between paedomorphic and metamorphic individuals during the time interval dominated by the Medieval Warm Period. There is a consistent ratio of paedomorphic to metamorphic specimens through the entire 3000 years, demonstrating that not all life history characteristics of the population were significantly altered by changes in climate on this timescale.

**Conclusion:**

The fossil record of *Ambystoma tigrinum *we used spans an ecologically relevant timescale appropriate for understanding population and community response to projected climatic change. The population-level responses we documented are concordant with expectations based on modern environmental studies, and yield insight into population-level patterns across hundreds of generations, especially the independence of different life history characteristics. These conclusions lead us to offer general predictions about the future response of this species based on likely scenarios of climatic warming in the Rocky Mountain region.

## Background

The global mean temperature has risen 0.6°C in the last 100 years and is projected to increase between 1.4°C and 5.8°C over the next 100 years [1]. This rate of increase (in temperature per century) is greater than any measured in the climate record of at least the last 100,000 years [2-4]. Several types of response to this rapid climatic change have been proposed for vertebrates, including range shifts to track resource changes, local adaptation to new resources, local extinctions, and developmental plasticity [5,6]. Although these changes are difficult to observe on a seasonal or annual time scale, empirical evidence from long-term field studies and historical records exists for range shifts and phenological shifts, especially earlier breeding events (see [4,5,7,8]). Clearly, long-term monitoring and historic data sets are critical to identification of meaningful biological responses to climatic change [9,10]. However, such information is scarce, with quality ecological records confined to only the last 100 years, and often far less [11]. One unique way to address these problems is by using biologically relevant time series data provided by the fossil record. An ideal fossil record would span the last few hundred to few thousand years in order to detect changes over tens to hundreds of generations. Such a record would provide a temporal scale long enough to identify significant trends and enough resolution to reveal ecologically relevant population responses. Ultimately, this type of data will provide information fundamental for predictions of response to climatic change over the next century.

Amphibians are often considered sensitive indicators of environmental conditions [12-16]. Many amphibian species have a naturally complex life cycle that involves both aquatic and terrestrial environments, directly exposing individuals to changes in environmental temperature and moisture over a single lifetime. Amphibian skin and eggs are highly permeable, which makes them sensitive to small changes in chemicals in the environment [17]. Furthermore, they are ectothermic, enabling differences in temperature to have direct effects on amphibian body temperature and metabolic processes [13,18]. Thus, projected global climatic change is expected to have significant effects on amphibian communities [18,19], but these effects have not yet been specified. Our study system provides the data necessary to initiate such predictions.

Lamar Cave is an exceptionally rich paleontological site located in Yellowstone National Park (YNP), Wyoming, USA [20]. The fossils are primarily from a wood rat midden collection of raptor pellets and carnivore scats. The excavated fossil collection contains tens of thousands of bones encompassing mammals, amphibians, birds, reptiles, and fish, and spans the last 3300 years [21]. Recent investigations of the small mammal record of Lamar Cave show morphological and genetic responses that correlate well with past climatic events in the area [22,23]. Lamar Cave also contains thousands of amphibian bones.

In particular, the most abundant amphibian in Lamar Cave, the tiger salamander (*Ambystoma tigrinum*) is an excellent species with which to study population level responses to climatic change. Many studies show that *Ambystoma *demonstrates developmental growth plasticity in response to environmental conditions (e.g. [24-27]): fossils record this as changes in mean body size. In addition, *Ambystoma *is able to exploit alternate life histories in response to different environmental conditions [26,28-31]. *Ambystoma *may undergo an aquatic larval stage and then metamorphose into terrestrial adults or alternately, be paedomorphic, *i.e.*, achieve sexual maturity while remaining aquatic and retaining larval morphological features. *Ambystoma tigrinum *is unusual in that it retains the ability to metamorphose facultatively even after years in the aquatic form [31]. The different life histories are detectable in diagnostic characters preserved in the fossil record [32,33] of populations represented in Lamar Cave. Thus, the cave records the potential effects of climatic change on several life history characteristics of *A. tigrinum*, and is documented here for the first time at this important temporal scale.

Paedomorphosis in modern *A. tigrinum *depends on various environmental stimuli [24,34]. Several biotic factors influence paedomorphosis and metamorphosis including thyroid hormone levels, larval density, and competition with fish [24,28,30,31,35,36]. Although important, these biotic factors are often overwhelmed by the influence of temperature and moisture. Populations in cold environments show high frequency of paedomorphosis [31] and are often large in size [27]. Populations found in ponds of intermediate temperature often contain both paedomorphs and metamorphosing individuals. Likelihood of paedomorphosis is also closely linked to pond permanence, or perceived pond permanence [24,28,29,37,38]. Pond permanence is directly related to available moisture in the environment in the form of humidity, precipitation, or ground water.

Given the environmental forces that drive body size and metamorphic changes in *A. tigrinum*, we used a late-Holocene fossil record to track these traits through the last 3000 years. We analyzed trends within the context of known climatic change, and attempted to distinguish patterns of response that represent specific climatic periods through this time.

## Results

The total number of identified *Ambystoma *specimens from the excavation was 2850. The total minimum number of individuals (MNI) from the excavation was determined to be 99. The abundance, represented by MNI, is relatively low in Intervals A, B, D, and E. Interval C, which corresponds to the bulk of the MWP, contains 66% of the *Ambystoma *fossils, while the intervals before (D and E) and after the MWP (A and B) all contain lower *A. tigrinum *abundance based on MNI (Table 1).

**Table 1:** NISP and MNI of *Ambystoma tigrinum *fossils by time interval. The difference between the total MNI and the paedomorph plus terrestrial MNI is due to young larval MNI.

Interval	Total NISP	Total MNI	Paedomorph MNI	Terrestrial MNI
A	156	10	5	4
B	170	8	3	1
C	2074	65	24	19
D	324	9	2	2
E	126	7	3	3

**Figure 2:**
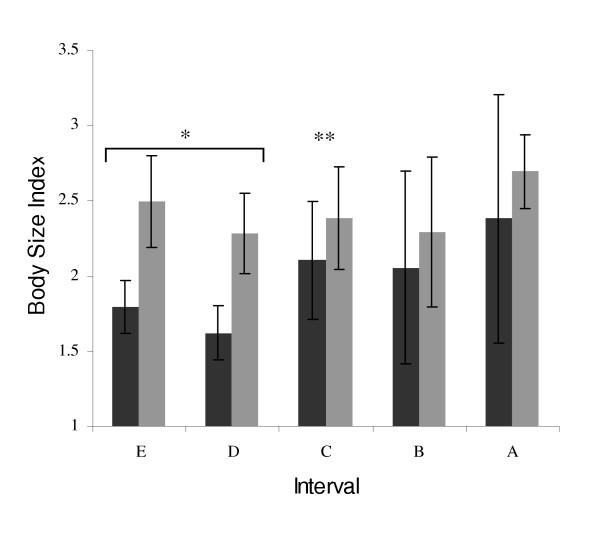
*Ambystoma tigrinum *Body Size Index (BSI) for paedomorphic (dark bars) and terrestrial adults (light bars) in time intervals. Error bars represent one standard deviation around mean. Bracket indicates significance when intervals D and E are pooled together. (* is p < .01 and ** is p < .002).

**Figure 3:**
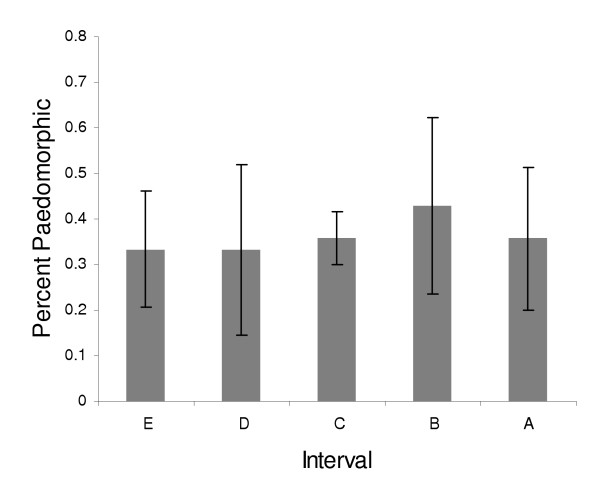
Percent paedomorphosis in time intervals. Percent paedomorphosis is expressed as paedomorphic minimum number of individuals (MNI) divided by adult MNI. Error bars represent one standard deviation around mean.

The body size index (BSI) measurements for the paedomorphs and the terrestrial adults do not show significant changes through time within an age class (two-way ANOVA, p > .10). However, the data do show variability through time that may be a measure of changes in the population, even if it is not a statistically significant deviation. To identify potentially biologically relevant trends between age class BSI's and known climatic periods, the terrestrial adult BSI was compared to the paedomorph BSI. There is a significant difference between the paedomorphic and terrestrial adults when analyzed by interval (ANOVA, p = .021). Comparisons within intervals indicate a significant difference in BSI between paedomorphic and terrestrial adults within interval C (t-test, p < .002; Figure 2). Within interval comparisons of BSI for intervals D and E do not show significant differences between paedomorphic and terrestrial adults at the .01 level (t-test, Bonferroni corrected significance level p = .01; interval D, p = .019; interval E, p = .012). However, lack of power may be due to small sample size. To further explore the difference in BSI between paedomorphic and terrestrial adults in the time period prior to interval C, we pooled intervals D and E, and find a significant difference in BSI (t-test, p < .001; Figure 2) We also pooled intervals A and B but did not find a significant difference in BSI (t-Test p >> .10) The percent paedomorphosis does not show significant variation through time (Figure 3; p >> .10).

## Discussion

An assumption made in interpreting the fossil record is that the specimens are representative of the sample population. Another caveat is that the common characteristics of the population also will occur in the fossil record if there are no effective sampling biases [21]. With this in mind, we expect changes in characteristics of the fossil specimens to represent changes in the sample population. Much of the fossil record for *A. tigrinum *through the last 3000 years indicates relatively conservative changes in population trends in morphotype, but nevertheless paedomorphic and metamorphic individuals are found throughout the deposit. Because there were no absolute shifts to a single life history strategy (i.e. paedomorphosis) within the sampled population over a relatively long ecological time period, it is likely that the two distinct life history strategies were maintained during short-term climatic fluctuations of the late Holocene. Thus, we conclude according to this case study that although change in frequency of paedomorphosis in a population occurs in relation to different environmental conditions over one generation, on the time scale of 100 to 1000s of years, if there is short-term climate variability little overall change would be expected in a population, even with coarsely distinguished climatic events.

Our results provide an opportunity to determine how the local population of *A. tigrinum *responded to the largest climatic anomaly in the Yellowstone region over the last 3000 years, the Medieval Warm Period (MWP) [39]. The MWP in Yellowstone is characterized by a warm and dry climate [40] and occurred approximately 1150 to 650 ybp [39]. These conditions suggest low pond permanence, or rapid drying, which could lead to shallower ponds that provide less protection from predators. The several ponds in the vicinity of Lamar Cave are all fishless glacial kettles with approximately the same water depth. Total *A. tigrinum *fossil abundance over the last 3,000 years shows a marked increase during Interval C, which corresponds to the MWP. Due to the deposition of this fossil assembly, fossil abundances reflect an overall increase in salamander abundance, an increase in successful predation and collection, or a combination of both factors. However, prior study on taphonomic biases potentially at work in this cave accumulation find no evidence for a change in predators, a change in collection radius, or an adjustment in collection vectors [21].

The periods of higher effective moisture may provide deeper, more permanent ponds that would offer greater protection from aerial predators, but we cannot conclude that such a sampling bias is the only factor affecting fossil abundance. The significant difference in BSI between paedomorphic and terrestrial adults during interval C, along with the increase in abundance, is evidence for warm climatic conditions that allows indeterminate growth of a terrestrial ectotherm to continue. This agrees with expected changes in *A. tigrinum *populations during the warming based on modern studies [41,42].

Our results also demonstrate that a population of *A. tigrinum *can show changes in average body size without changes in the percentage of different morphologies. In the oldest intervals, E and D, paedomorphs appear to have been smaller (based on BSI) than in other intervals, while the percent paedomorphic remained relatively constant. This suggests a favorable terrestrial environment where metamorphosing individuals continue to grow following the transition from the aquatic environment. This terrestrial growth will be most facilitated by warm conditions and abundant food. The available climate records show these relatively long time periods as variable, with periods of high and low effective moisture [43-45].

There are a variety of confounding factors that complicate our interpretations. First, although the excavation was remarkably rich and the number of identified salamander specimens quite large, our conservative use of MNI reduced our sample size. Our use of time intervals (A-E), while justified based on our own ^14^C chronology, exacerbated time-averaging [20]. In addition to impacts on the fossils, the intervals create a potential time averaging error for the climatic record. In fact, several intervals span more than one particular climatic period, while certain major climatic periods span more than one interval. Thus, although the fossil record does not indicate significant changes in certain traits, there may be larger variation in those traits that simply is not recorded in the fossil record or that is masked by the effect of time averaging. Also, the overall influence of the abiotic environment may have the most significant effect on *Ambystoma *metamorphosis, yet we cannot dismiss the role of certain biotic elements. Specifically, density-dependent effects are correlated with body size and metamorphosis, which may skew our interpretations. Larval density will impact resource competition, with higher competition driving metamorphosis. Patterns in larval individuals have not been documented here because of small sample sizes. Finally, likelihood of paedomorphosis may have a significant genetic control, which we do not discount here. Our results support the maintenance of alternate, but coexisting, life history strategies suited to selection pressures in variable environments.

## Conclusion

The late Holocene *Ambystoma *fossils from Lamar Cave provide a unique opportunity to examine the important ecological and evolutionary implications of changes in life history characteristics in response to climatic change. We present a record of change in a population of tiger salamanders over the last 3000 years. Significant perturbation in the environment is possible under future climate change scenarios, and the role of local populations in the terrestrial or aquatic system may be altered. Warmer and drier climate scenarios as predicted for the Yellowstone region [46] would likely create less permanent aquatic environments and select for populations with primarily metamorphosing individuals, against the retention of paedomorphosis. This scenario would decrease the vertebrate biomass in the aquatic system as well as reduce the predatory pressure on aquatic *Ambystoma *food sources. Such changes to the ecological system could result in unexpected biological feedbacks. Also, higher percentages and rates of metamorphosis would increase gene flow between populations. This, combined with probable decreases in sizes of populations, has the potential to alter the overall genetic diversity in the meta-population over time, perhaps reducing the ability of the species to respond to further perturbation of the system.

## Methods

### Study site and fossil collection

The Greater Yellowstone Ecosystem (GYE) is often considered one of the last intact, temperate ecosystems in the world. This ecosystem contains all native mammals and few exotics, and is thought to be functioning in a relatively natural state [47]. The GYE is located in northwestern Wyoming, and contains portions of southern Montana and eastern Idaho (center of park: 44° 36' 53.25"N Latitude, 110° 30' 03.93" W Longitude). The core of the GYE is Yellowstone National Park (YNP), which was established as the world's first national park in 1872. The preservation of this park means that we are able to extend current ecological conditions to the recent past.

The *A. tigrinum *fossils used in this analysis were excavated from Lamar Cave, a paleontological site in YNP. The details of the excavation and stratigraphy are described elsewhere [20]. Isotopic analysis has shown the sampling radius of the cave to be within 8 km (with 95% confidence) [48]. Within this radius there are at least 19 fishless, modern ponds of generally similar permanence that are potential habitat for *A. tigrinum*. The *A. tigrinum *samples are most likely from predation in these ponds and surrounding lands. The current study analyzes fossils obtained from 15 of the 16 stratigraphic levels from the excavation (level 11 did not contain any *Ambystoma *specimens). For the analyses the levels were pooled into five intervals, labeled A-E (youngest to oldest). This aggregation was based on 95% confidence limits around the radiocarbon dating of the intervals [20].

**Figure 1:**
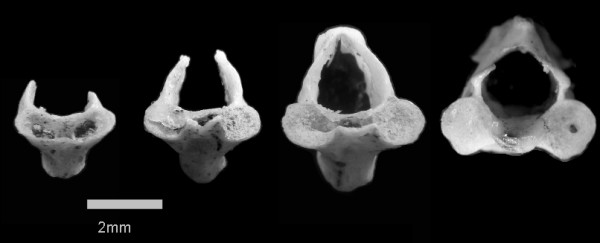
Four age classes of cervical *Ambystoma tigrinum *vertebrae. Scale is in millimeters. Left to right: Young Larval show an open (unfused) neural arch and open centrum with little or no ossification; Young Terrestrial are characterized by an open neural arch and constricted, or partially fused, centrum with little ossification; the Paedomorphs are typified by a fused neural arch and an open centrum with some ossification present; the Old Terrestrial are described by a fused neural arch and a closed, or fused, centrum with visible ossification.

Easily identified *A. tigrinum *fossils include femora, humeri, vertebrae, and various skull bones. We used the fossil vertebrae because of their abundance (N = 2850) and because they record metamorphic state. All vertebrae were identified, but for the purposes of this study only the first cervical and sacral vertebrae were used. Because these particular vertebrae are unique to every skeleton, they are useful in determining the minimum number of individuals from a locality [49]. The fossils were grouped within each stratigraphic layer into four morphologically distinct classes: Young Larval, Paedomorphic, Young Terrestrial, or Old Terrestrial. The developmental stage and age of each individual was determined from diagnostic characteristics of the neural arch and centrum [32,33]. Specifically, the Young Larval had an open (unfused) neural arch and open centrum with little or no ossification; the Young Terrestrial were characterized by an open neural arch and constricted, or partially fused, centrum with little ossification; the Paedomorphic were typified by a fused neural arch and an open centrum with some ossification present; the Old Terrestrial were described by a fused neural arch and a closed, or fused, centrum with visible ossification (Figure 1).

Abundance was determined by a standardized minimum number of individuals (MNI) [49]. The MNI was taken as the larger of the two values for sacral or cervical vertebrae (axis), since the *Ambystoma *skeleton contains only one of each of these elements. The abundance levels were then standardized by dividing by the MNI of the wood rat, *Neotoma cinerea*. Unlike other common small mammals found at this site, wood rats show a constant relative abundance [20]. This pattern is consistent with a broad habitat preference for this species, and is especially important because the wood rat is the main collection agent of the Lamar Cave fossils. Their relative evenness thus indicates taphonomic constancy of the cave [21], which is corroborated with isotopic analyses [48].

Because plasticity in growth rate cannot be directly measured in the fossil record, it is inferred from body size in different age classes. The centrum length and anterior width of each specimen were measured with electronic calipers. A body size index (BSI) was created for each specimen by dividing the centrum length by the anterior centrum width [32].

Percent paedomorphosis by time interval was determined by dividing the standardized MNI of paedomorphic vertebrae by the standardized MNI of all adult vertebrae, defined as Old Larval, Young terrestrial, and Old Terrestrial morphs. Thus, we calculate abundance, mean body size, and percent paedomorphosis, each as potentially independent responses of the salamander population to the abiotic environment around Lamar Cave.

## Authors' contributions

EAH designed the study and collected the specimens. WL collected data. JEB analyzed data.
